# Snakebite associated thrombotic microangiopathy: a protocol for the systematic review of clinical features, outcomes, and role of interventions

**DOI:** 10.1186/s13643-019-1133-2

**Published:** 2019-08-22

**Authors:** Tina Noutsos, Bart J. Currie, Geoffrey K. Isbister

**Affiliations:** 1 Menzies School of Health Research, Charles Darwin University, Darwin, NT Australia; 2 College of Medicine and Public Health, Flinders University, Adelaide, SA Australia; 3 Division of Medicine, Royal Darwin Hospital, Darwin, NT Australia; 4 Clinical Toxicology Research Group, University of Newcastle, Newcastle, NSW Australia

**Keywords:** Snake, Envenoming, Thrombotic, Microangiopathy, Schistocytes, Haemolysis, Anaemia, Acute kidney injury, Plasmapheresis

## Abstract

**Background:**

Thrombotic microangiopathy is an uncommon complication of snake envenoming associated with a subset of snakebite cases presenting with venom-induced consumption coagulopathy. It presents with microangiopathic haemolytic anaemia and thrombocytopenia. Available studies are predominantly small retrospective observational studies only. Renal end organ injury appears common. It has variably been likened to either thrombotic thrombocytopenic purpura or atypical haemolytic uraemic syndrome. Many studies report acute kidney injury, with dialysis required in a subset of patients. Some studies suggest a role for treatment with plasmapheresis. Interpretation of the literature is complicated by variable nomenclature and historically poor case definitions of both venom-induced consumption coagulopathy and thrombotic microangiopathy associated with snake envenoming.

**Methods:**

The main objective of this systematic review is to synthesise the worldwide experience of snakebite-associated thrombotic microangiopathy with respect to its features and outcomes, and collate any evidence for the role of plasmapheresis as treatment. A predetermined case definition of confirmed or suspected snakebite with either blood film evidence of microangiopathic haemolytic anaemia or histological evidence of thrombotic microangiopathy will be used. This case definition will determine the search terms and study inclusion criteria. Relevant studies will be identified by electronic database, published conference abstracts, and grey literature search. This systematic review will be performed and reported according to the Preferred Reporting Items for Systematic Reviews and Meta-Analyses checklist. The quality of included studies will be assessed by a proposed tool for evaluating the methodological quality of case reports and case series. Results will be reported by a descriptive synthesis with a focus on the presenting features; outcomes of acute kidney injury, dialysis-free survival, other end organ damage, and overall survival; and any evidence of a role for treatment with plasmapheresis. The quality of accumulated evidence will be assessed via the Grading of Recommendations, Assessment, Development, and Evaluations framework.

**Discussion:**

It is anticipated that eligible studies will consist of small observational studies. Evidence gathered from this review will provide the first broader understanding of the clinical features, outcomes, prognosis, and management of snakebite-associated thrombotic microangiopathy.

**Systematic review registration:**

PROSPERO CRD42019121436

**Electronic supplementary material:**

The online version of this article (10.1186/s13643-019-1133-2) contains supplementary material, which is available to authorized users.

## Background

Snakebite causes a variety of clinical sequelae. Local symptoms and signs include local bite site pain, swelling, and tender regional lymphadenopathy. Non-specific systemic symptoms include nausea, vomiting, headache, diaphoresis, abdominal pain, and diarrhoea. Systemic envenoming can cause major toxin syndromes, including neurotoxicity, myotoxicity, and coagulopathy. Different snake species are associated with characteristic patterns of these toxicities. These differences are conferred by their variable venom composition of non-enzymatic and enzymatic toxins [1], including cytotoxins, myotoxins, neurotoxins, and haemotoxins. Haemotoxins are associated with viper and Australian elapid envenomings, and with cardiovascular and haemostatic effects, including hypotension, cardiovascular collapse, coagulopathy, and bleeding [2].

The most common haemotoxin-mediated coagulopathy caused by snake envenoming is a venom-induced consumption coagulopathy (VICC), characterised by prolonged clotting times, hypofibrinogenaemia, and a raised d-dimer [2–5]. VICC resolves at a rate consistent with synthesis of new clotting factors, after the toxins are neutralised. Its main complication is bleeding [5]. VICC is distinct from disseminated intravascular coagulopathy (DIC) [3]. Whilst VICC meets the definitions of commonly used diagnostic scoring systems for DIC, the differing pathophysiology, outcomes, and prognosis of VICC compared to DIC clearly differentiate it [3, 6, 7]. A small subset of snakebite patients with VICC develop a thrombotic microangiopathy (TMA) [3, 8–17]. The reported cases of snakebite-associated TMA present with a delayed phase thrombocytopenia and a haemolysis in keeping with microangiopathic haemolytic anaemia (MAHA) [11]. There appears to be a predilection for renal end organ injury [3, 11]. The long-term outcomes and best treatment of snakebite-associated TMA are unclear, with most published studies being isolated case reports, small case series, or other small retrospective observational studies.

The TMA syndromes are a group of disorders with the unifying pathognomonic hallmark of vascular small vessel wall damage with microthrombosis [18–20]. There is a resultant MAHA with mechanical red cell fragmentation which manifests as haemolysis with circulating red cell fragments (schistocytes) on examination of the blood film [18, 21]. Thrombocytopenia together with MAHA is sufficient for the diagnosis, and tissue biopsy for confirmation by the finding of TMA is only occasionally performed [18, 20]. Corroborating other signs of haemolysis include the presence of anaemia, a raised lactate dehydrogenase (LDH), unconjugated hyperbilirubinaemia, and lowered haptoglobin [20]. However, these are markers of haemolysis of any cause and are not specific to MAHA. The main complication of TMA is vaso-occlusive end organ injury [18]. This may be multi-organ in nature (commonly seen for example with DIC), or for certain forms of TMA, it may be more organ-specific: neurological for thrombotic thrombocytopenic purpura (TTP) [18, 21] and renal for haemolytic uraemic syndrome (HUS) [18, 20]. The aetiologies of TMA disorders differ. DIC is caused by complex mechanisms which culminate in generalised microvascular fibrin deposition due to thrombin generation triggered by a tissue factor stimulus in the intravascular space [6]. TTP is mediated by either a hereditary- or an autoimmune antibody-mediated deficiency of a disintegrin and metalloproteinase with a thrombospondin type 1 motif, member 13 (ADAMTS-13) [19]. Most cases of HUS are caused by Shiga toxin producing or enterohaemorrhagic *Escherichia coli* (typical HUS), or acquired or hereditary dysregulation of the alternative complement pathway in atypical HUS (aHUS) [20]. The TMAs are heterogeneous not only in both their clinical presentation and aetiology, but also in their best treatments and long-term outcomes.

Snakebite-associated TMA has been variably compared to both TTP and aHUS [11, 13, 22]. Plasmapheresis has a clearly established role in dramatically preventing mortality in TTP [11, 19, 21, 23]. Some published cases of snakebite-associated TMA with acute kidney injury (AKI) have reported successful treatment with plasmapheresis, with normalisation of renal function [11, 13]. Other published cases of snakebite-associated TMA with AKI report that the renal end organ damage resolves with renal replacement therapy alone [11]. Any association with respect to the pathophysiology or long-term outcomes of snakebite TMA with either aHUS or TTP is not established [3, 11]. The potential role of plasmapheresis in the treatment of snake TMA treatment is also unknown.

Interpretation of the published literature on snakebite TMA and VICC more broadly is complicated by historical differences and inconsistencies in nomenclature. Many studies use a variety of terms, often not overtly defined, including DIC, defibrination, and intravascular haemolysis [3, 5, 9, 12, 24, 25]. VICC is commonly but erroneously reported as DIC, and there is a lack of consistency in the case definition for snakebite-associated TMA. Various and often ill-defined haematological abnormalities together with AKI are also commonly reported in the setting of snakebite [26]. Complicating this further, AKI in snakebite is common and has a number of other possible causes including direct venom toxicity, shock, and myotoxicity with secondary rhabdomyolysis [9, 12, 27]. This leads to difficulties in establishing which cases are true TMA versus the more typical VICC of snake envenoming, and which represent other causes of snakebite with AKI and coincidental haematological findings. These challenges make the incidence, features, natural history, outcomes, and best treatment strategy for snakebite TMA difficult to elucidate.

The objective of this systematic review is to synthesise the features, outcomes, and evidence of any role for intervention with plasmapheresis, for snakebite-associated thrombotic microangiopathy, using a predetermined case definition. We hope to inform best practice via a descriptive synthesis of its features, outcomes, and any evidence for or against current reported approaches to its management.

## Methods/design

### Research objectives

The aims of this systematic review will be as follows:

- To identify all relevant studies in TMA associated with snakebite via a prespecified evidence-based case definition composed of the presence of confirmed snakebite, with findings of TMA on either histological tissue biopsy or autopsy, or blood film findings of the schistocytosis of MAHA.
- To perform a descriptive synthesis of snakebite-associated TMA baseline characteristics, presenting clinical and laboratory features, outcomes of AKI, dialysis-free survival, other end organ damage, overall survival, and the intervention of plasmapheresis.

### Research hypotheses and assumptions

The hypotheses and assumptions prior to conducting the systematic review are as follows:

- Renal end organ damage is the predominant organ affected by TMA in snakebite.
- There will be differences in snake genera and species by geographical location, time to treatment, access to treatment options, and outcome of mortality influenced by variable resource limitations.
- The event is rare, and only small observational studies (case reports, case series) will be eligible for inclusion.
- Data reporting will be variable in standardisation or classification of outcome measures such as AKI, VICC, and related coagulation testing.

### Systematic review design and registration

This systematic review and meta-analysis will be performed according to the Preferred Items for Systematic review and Meta-Analysis (PRISMA) checklist [28] (Additional file 1). The review protocol is registered with PROSPERO (CRD42019121436).

### Population, interventions, outcomes, and study design

A Population Intervention Context Outcomes Study Design (PICOS) table is shown in Table 1.

**Table 1:** Population Intervention Context Outcomes Study Design (PICOS)

Population	Humans (all ages) sustaining a snakebite with TMA features as defined by either histological biopsy or definite blood film features of microangiopathy (schistocytes or red cell fragments)
Intervention	Plasmapheresis
Context	Attending for medical care in any setting worldwide
Primary outcome	Dialysis-free survival
Secondary outcomes	Acute kidney injury
	Other end organ damage
	Overall survival
Study design	All studies reporting original data

### Identification of studies: information sources and search strategy

Potentially eligible studies will be identified by electronic database search of PubMed, Medline via EBSCO, and the Cochrane library. Other potential sources of studies will be sought via relevant published conference abstracts and grey literature search of the Grey Matters checklist, Google Scholar, opengrey.eu, grelit.org; GreyNet, Grey Literature Report, and BIOSIS Previews.

A comprehensive and inclusive search strategy has been designed. The full search strategy for PubMed is available in Additional file 2. Relevant search terms used will be controlled vocabulary Medical Subject Heading (MeSH) terms whenever possible, or alternatively free text search terms. Search terms will be combined with Boolean operators.

To allow for the greatest sensitivity, PubMed searches will utilise MeSH term explosion, and Medline search mode expanders will be used to apply related words and search within the full text of articles and apply equivalent subjects. Searches will be performed for all publication types. Results in the search will be limited to human studies, with no publication restriction based on study language, age, or design. Both quantitative and qualitative studies will be included. Studies will not be limited by place or country of publication. Both published and unpublished literature including unpublished studies, reports, conference abstracts, dissertations, and conference papers will be considered for eligibility.

### Eligibility

Inclusion and exclusion criteria are designed to enable an inclusive review and to best capture all worldwide cases meeting the predetermined case definition of snakebite-associated TMA.

Studies will be included if they meet the following inclusion criteria based on the study population of interest:

- Human cases of all ages
- Suspected or confirmed snakebite of any species
- Definite features of thrombotic microangiopathy as evidenced by either of the following:
  - Blood film red cell fragmentation (schistocytosis)
  - Tissue biopsy or autopsy histological findings of TMA

The following exclusion criteria will be used:

- Studies reporting in vitro data only
- Review studies not reporting original data
- Studies reporting cases already described in other original publications by the same author, with complete overlap of reporting

### Study selection

Each retrieved publication title and abstract will be screened for relevance by the first author (TN). Articles in any language will be included provided an English language abstract is available to screen for potential relevance and a full-text translation is possible to source. Relevant full-text articles will then be screened against inclusion and exclusion criteria by two independent reviewers for eligibility. Any disagreement between the two reviewers regarding eligibility will be resolved by discussion and consensus. The reference sections of all full reviewed journal articles will be manually searched for relevant articles that may have been missed. Studies excluded from the review on the basis of not reporting original data will have their reference bibliography manually searched for potentially relevant publications. Study selection will be reported using the PRISMA flow diagram (Additional file 3).

### Quality assessment of risk of bias in included studies

Methods used for assessing risk of bias will be done at a study rather than outcome level. It is assumed that most studies eligible for inclusion will be case reports, case series, or small observational studies. With the known risk of bias associated with small uncontrolled study designs, the framework for evaluation of risk of bias to be used for this systematic review is that proposed by Murad et al. [29] (Table 2), against the domains of study selection method, ascertainment for exposure and outcome, causality, and reporting. Two independent reviewers will assess risk of bias, and any disagreement will be resolved by discussion and consensus. The implications of this risk of bias assessment will be explicitly addressed within the discussion of the systematic review’s findings.

**Table 2:** Evaluation tool for methodological quality of case reports and case series, adapted from Murad et al [29]

Domain	Relevant leading explanatory questions
Selection	1. Does the study selection method of the case(s) enable a representation of the whole experience of the investigator or reporting centre or is the selection method unclear to the extent that other patients with similar presentation may not have been reported?
Ascertainment	2. Was the exposure of snakebite envenoming adequately ascertained?
	3. Was (were) the outcome(s) adequately ascertained?
Causality	4. Were alternative causes that may explain the observation(s) ruled out?
	5. Was follow up long enough for outcomes to occur?
Reporting	6. Is the case described with sufficient detail to allow other practitioners to make inferences relating to their own practice?

### Data extraction and coding

Data collection for studies meeting inclusion criteria after full-text review will be by a data extraction spreadsheet. This will record baseline demographics, snake genera and species, timing and signs of envenomation, serial haemoglobin, platelet, coagulation and creatinine laboratory results, use and timing of interventions of plasmapheresis, antivenom, supportive treatment including blood product transfusion, and the outcomes of acute kidney injury, renal recovery including dialysis-free survival, other end organ injury, and overall survival. The data extraction spreadsheet will be piloted on three studies and then reviewed for relevance before proceeding to further full-text data extraction. Two independent reviewers will perform data extraction. Any discrepancy will be resolved by discussion and consensus. Wherever possible, the original investigator will be contacted by email to request any missing data. If unsuccessful, the limitations of any method used to deal with missing data will be explicitly stated, together with the potential impact of the missing data within the discussion of the review’s findings.

Data where possible will be transformed into SI units and where relevant will be unified and categorised as per Additional file 4. Laboratory data on the consumption coagulopathy of snake envenomation will be coded where possible according to presence of VICC and classification of VICC as either complete or partial [5]. If reported data allow, the renal outcome of AKI will be coded according to the Kidney Disease: Improving Global Outcomes (KDIGO) AKI classification [30]. The course and outcomes of renal recovery will be categorised by acute kidney disease and chronic kidney disease stage [31], and freedom from dialysis. It is foreseen that urine output records may not be available in which case classification will be done based on creatinine, presence of anuria, and initiation of renal replacement therapy alone.

Larger studies reporting only a subgroup of patients meeting the inclusion criteria for this study will have data extracted relating to the defined study population of interest. Given the assumption that most eligible studies will be case reports, case series, or small observational studies, it is possible that line by line individualised patient data may be readily extractable for each eligible study.

### Synthesis and statistical analysis of results

A descriptive synthesis of the findings will outline the characteristics of the included studies, baseline demographics of cases, clinical and laboratory features, and summarised data on outcomes and interventions used. A quantitative synthesis is not planned. A subgroup analysis of snakebite-associated TMA requiring dialysis will be undertaken by narrative synthesis of the outcomes of dialysis-free survival for those treated with plasmapheresis versus best supportive care only. The level of agreement between the two independent reviewers for study selection, data extraction, and risk of bias assessment will be reported by Cohen’s kappa statistic. The strength of confidence in the cumulative evidence for this systematic review will be addressed with reference to the Grading of Recommendations Assessment, Development, and Evaluation (GRADE) working group framework [32] within the discussion of the systematic review findings.

### Dissemination plans

A paper of the systematic review will be submitted for publication to a leading journal in the field of clinical toxicology or haematology.

## Discussion

Snakebite-associated TMA is a rare and poorly understood phenomenon. Existing literature consists of small observational studies with little to guide an understanding of its natural history, outcomes, and treatment. This is complicated by published studies which use varied and sometimes non-defined descriptors for both the coagulopathy and thrombotic microangiopathy of snake envenomation. Published cases have been likened to either aHUS or TTP. Plasmapheresis has been used in some studies with perceived benefit. Other studies report that the renal end organ damage is self-limiting.

This systematic review will provide the first descriptive synthesis of the international experience of snakebite-associated TMA. We will use a predefined case definition of snakebite-associated TMA, which will help inform an understanding of the clinical features and typical outcomes of this uncommon phenomenon. The most likely limitation to this systematic review will be the quality of included studies, which will likely be predominantly small observational studies.

We aim for this systematic review to help guide best intervention strategies including elucidating any role for plasmapheresis, which, whilst having a clear role in the prevention of mortality and morbidity in some types of TMA, is a resource-intense intervention which also carries some adverse risk.

## Additional files

Additional file 1: Preferred Items for Systematic review and Meta-Analysis (PRISMA) checklist. (DOCX 33 kb)

Additional file 2: Full search strategy for PubMed. (DOCX 15 kb)

Additional file 3: PRISMA flow diagram. (DOCX 49 kb)

Additional file 4: Methods to unify and categorise data. (DOCX 16 kb)

## Data Availability

Not applicable.

## Abbreviations

ADAMTS-13: A disintegrin and metalloproteinase with a thrombospondin type 1 motif, member 13
aHUS: Atypical haemolytic uraemic syndrome
AKI: Acute kidney injury
DIC: Disseminated intravascular coagulopathy
HUS: Haemolytic uraemic syndrome
INR: International normalised ratio
KDIGO: Kidney Disease: Improving Global Outcomes
LDH: Lactate dehydrogenase
MAHA: Microangiopathic haemolytic anaemia
MeSH: Medical Subject Headings
PICOS: Population Intervention Context Outcomes Study Design
PRISMA: Preferred Items for Systematic review and Meta-Analysis
TMA: Thrombotic microangiopathy
TTP: Thrombotic thrombocytopenic purpura
ULN: Upper limit of normal
VICC: Venom-induced consumption coagulopathy
