# Supplementary material for: Snakebite associated thrombotic microangiopathy: a protocol for the systematic review of clinical features, outcomes, and role of interventions
Source: Syst Rev. 2019 Aug 22;8:212. doi: 10.1186/s13643-019-1133-2 (PMC6706936; doi:10.1186/s13643-019-1133-2)
Supplement: Supplementary file 2 — Full search strategy for PubMed. (DOCX 15 kb) [file 13643_2019_1133_MOESM2_ESM.docx]

**Additional file 2: PubMed search strategy:**

#1 snakes [MeSH Terms]

#2 snake bites [MeSH Terms]

#3 venoms [MeSH Terms]

#4 #1 OR #2 OR #3

#5 thrombotic microangiopathies [MeSH Terms]

#6 thrombosis [MeSH Terms]

#7 erythrocytes [MeSH Terms]

#8 schistocyt* [Text Words]

#9 anemia, hemolytic [MeSH Terms]

#10 hemolysis [MeSH Terms]

#11 “red cell” [Text Words]

#12 fragment* [Text Words]

#13 #11 AND #12

#14 kidney diseases [MeSH Terms]

#15 multiple organ failure [MeSH Terms]

#16 #5 OR #6 OR #7 OR #8 OR #9 OR #10 OR #13 OR #14 OR #15

#17 #4 AND #16
