# Supplementary material for: Snakebite associated thrombotic microangiopathy: a protocol for the systematic review of clinical features, outcomes, and role of interventions
Source: Syst Rev. 2019 Aug 22;8:212. doi: 10.1186/s13643-019-1133-2 (PMC6706936; doi:10.1186/s13643-019-1133-2)
Supplement: Supplementary file 3 — PRISMA flow diagram. (DOCX 49 kb) [file 13643_2019_1133_MOESM3_ESM.docx]

**Additional file 3: PRISMA study flow diagram**

Derived from: Moher D, Liberati A, Tetzlaff J, Altman DG, The PRISMA Group (2009). Preferred Reporting Items for Systematic Reviews and Meta-Analyses: The PRISMA Statement. PLoS Med 6(7): e1000097. doi:10.1371/journal.pmed1000097

Titles/abstract records identified through database searching

(n = )

Additional titles/abstract records identified through other sources
(n = )

## Identification

Titles/abstract records after duplicates removed

(n = )

## Screening

Titles/abstract records screened
(n = )

Title/abstract records excluded as not relevant

(n = )

## Eligibility

Full-text articles excluded from analysis with reasons

(n = )

Potentially relevant full-text articles assessed for eligibility
(n = )

Studies included in data extraction

(n = )

## Included

Studies included in systematic review

(n = )
