# Supplementary material for: Snakebite associated thrombotic microangiopathy: a protocol for the systematic review of clinical features, outcomes, and role of interventions
Source: Syst Rev. 2019 Aug 22;8:212. doi: 10.1186/s13643-019-1133-2 (PMC6706936; doi:10.1186/s13643-019-1133-2)
Supplement: Supplementary file 4 — Methods to unify and categorise data. (DOCX 16 kb) [file 13643_2019_1133_MOESM4_ESM.docx]

**Additional file 4: Methods to unify and categorise data**

| **Study variable or outcome** | | **Plan to unify or categorise data** |
| --- | --- | --- |
| **MAHA** | |  |
|  | Haemoglobin nadir | Lowest recorded haemoglobin in g/L |
|  | Time to haemoglobin nadir | Time to lowest recorded haemoglobin in g/L from snake bite (hours and minutes) |
|  | Platelet nadir | Lowest recorded platelet count (10^9^/L) |
|  | Time to platelet nadir | Time to lowest recorded platelet count (10^9^/L) from snake bite (hours and minutes) |
|  | Maximum LDH | Maximum LDH (U/L) and as ratio of ULN for reported reference range (times ULN) |
|  | Time to maximum LDH | Time to maximum LDH (U/L) from snake bite (hours and minutes) |
| **VICC** | |  |
|  | Complete VICC | Undetectable fibrinogen by Clauss method; OR INR>3 and D-dimer at least 10 times assay ULN or >2.5mg/L |
|  | Partial VICC | Low but detectable fibrinogen, OR raised D-dimer AND maximum INR<3.0 |
| **KDIGO AKI stage^a^** | |  |
|  | Stage 1 | Day 1-7: creatinine 1.5-1.9 times baseline or ≥26.5 µmol/L increase; OR urine output <0.5ml/kg/h for 6-12 hours |
|  | Stage 2 | Day 1-7: creatinine 2.0-2.9 times baseline; OR urine output <0.5ml/kg/h for ≥12 hours |
|  | Stage 3 | Day 1-7: creatinine 3.0 times baseline or increase to ≥353.6 µmol/L; OR initiation of RRT; OR urine output <0.3ml/kg/h for ≥24 hours or anuria for ≥12 hours; OR in patients <18 years old, decrease in eGFR to <35ml/min/1.73m^2^ |
| **ADQI ACD stage^b^** | |  |
|  | Stage 1 | Continuation of AKI stage 1 between day 8-90 |
|  | Stage 2 | Continuation of AKI stage 2 between day 8-90 |
|  | Stage 3 | Continuation of AKI stage 3 between day 8-90 |
| **CKD stage** | |  |
|  | Stage 1 | eGFR ≥90ml/min/1.73m^2^ AND albuminuria, haematuria, a pathological abnormality or a structural abnormality |
|  | Stage 2 | eGFR 60-89ml/min/1.73m^2^ |
|  | Stage 3a | eGFR 45-59ml/min/1.73m^2^ |
|  | Stage 3b | eGFR 30-44ml/min/1.73m^2^ |
|  | Stage 4 | eGFR 15-29ml/min/1.73m^2^ |
|  | Stage 5 | eGFR <15ml/min/1.73m^2^ |
| ^a^KDIGO AKI staging is by most concerning feature of either creatinine rise, urine output or use of renal replacement therapy, during day 1 to 7 from renal insult. ^b^ADQI ACD staging is defined as ongoing AKI between day 8 to 90. MAHA: microangiopathic haemolytic anaemia; LDH: lactate dehydrogenase; VICC: venom induced consumption coagulopathy; INR: International normalised ratio; ULN: upper limit of normal; KDIGO: Kidney Disease: Improving Global Outcomes; AKI: acute kidney injury; ADQI: Acute Disease Quality Initiative; ACD: acute kidney disease; CKD: chronic kidney disease; eGFR: estimated glomerular filtration rate | | |
